# Controllable Hartman effect by vortex beam in a one dimensional photonic crystal doped by graphene quantum dots

**DOI:** 10.1038/s41598-023-29891-4

**Published:** 2023-02-20

**Authors:** Saeideh Kevin, Mostafa Sahrai, Seyyed Hossein Asadpour

**Affiliations:** 1 Faculty of Physics, University of Tabriz, Tabriz, Iran; 2 School of Physics, Institute for Research in Fundamental Sciences (IPM), P.O. Box 19395-5531, Tehran, Iran

**Keywords:** Optics and photonics, Physics

## Abstract

The Hartman effect is studied in a one dimensional photonic crystal doped with graphene quantum dots. It is shown that the Hartman effect can be switched from negative to positive by increasing the Rabi-frequency of the controlling field and also by manipulating the relative phase of the applied fields. The effect of the vortex beam on the Hartman effect is also presented. We show that the orbital angular momentum (OAM) and the azimuthal phase of the vortex beam do not affect the probe filed transmission while they change the Hartman effect from positive to negative.

## Introduction

Quantum mechanics predicts tunneling of a photon through a classically forbidden barrier^1–3^. It takes a limited time for a wave packet to tunnel through a barrier. This period of time directly relates to the length of the barrier, i.e. the larger barrier width, the longer tunneling time^4^. When the length of the barrier becomes large enough, the tunneling time becomes independent of the barrier length and tends to a constant^5^. This phenomenon, which implies the superluminal light propagation, is referred to as the Hartman effect^6–8^. The Hartman effect was investigated in various proposals including spin waves^9^, field emission^10^, graphene systems^11^, quantum networks^12^.

Multi-layer structures with periodic refractive indices such as one dimensional photonic crystals (1DPCs) are the proper media for controlling the behavior of the Hartman effect. These 1DPCs have important roles in optical devices that impresses the light–matter interaction such as the group velocity of a light pulse^13,14^, optical sensors^15^, biosensors^16,17^, temperature sensors^18^, wavelength demultiplexer^19^ and other numerous applications^18,20–25^. An important feature of the 1DPCs is the existence of the photonic band gap in its transmission spectrum. An electromagnetic field propagating through a 1DPC is evanescent when its frequency lies within the band gap^26,27^. The phase time of a propagating wave packet through a 1DPC with positive refractive indices is always positive, which is referred to as the positive Hartman effect and implies to subluminal light propagation^28^. However, in some media such as a wave guide^29^, the phase time can be negative, which corresponds to the negative Hartman effect. However, by introducing a defect layer doped with atoms or quantum dot structures, the Hartman effect can be controlled and even switched from negative to positive^30,31^. We investigated (with collaborators) the Hartman effect in 1DPC with a defect layer doped with three-level atoms^30^. It is found that the transmitted phase time can be switched from positive to negative by manipulating the Rabi-frequency of the applied controlling fields. We also demonstrated that the Hartman effect can be controlled by the rate of an incoherent pumping field and adjusting the relative phase of the applied fields^29^. In another proposal, we found that the Hartman effect can be controlled by the relative phase of the applied fields and also by choosing the proper incident angle of the probe field^31^. It is interesting to investigate the position dependent Hartman effect by the orbital angular momentum of the light beam. It is well-known that a light beam carries an orbital angular momentum (OAM), which is a highly efficient parameter for controlling the propagation of the light beam^32,33^. Allen et al.^34^ showed that a light beam with helical phase front carries a particular amount of OAM around the propagation axis. The only possibility to have a helical phase front in a light beam is the singularities along the center of the light beam. This does not possess any OAM, and does not exchange any energy with its surrounding. This kind of light beam, which carries a rotational current around a phase singularity, is called a vortex beam. One kind of these vortex beams is Laguerre–Gaussian cylindrical modes with a phase factor as $e^{il\Phi }$. Here, the parameters $l$ and $\Phi$ denote the topological charge of the vortex beam and the azimuthal phase, respectively. The topological charge determines the amount of OAM carried by each photon.

In the recent decades, graphene quantum dots have been interesting candidate for new applications due to their unique optical properties in various subjects such as nanoelectronics and condensed matter physics. As it is well known that some of the most significant properties of graphene quantum dots are their large transition dipole moments, flexibility in their design and controllable energy diagram^35,36^. In a recent study, a one-dimensional photonic crystal with a defect layer doped with graphene quantum dots has been utilized as an all-optical switch. It is shown that the existence of graphene quantum dots results in switching the subluminal to superluminal light propagation^37^. Furthermore, magneto-optical properties and layered structures of graphene quantum dots have attracted many scientists. Recently it is shown that an enhanced refractive index with suppressed absorption of a weak probe field in graphene nano structures under an external magnetic field can be obtained^38^.

In this paper, we investigate the propagation of a light pulse passing through the 1DPC doped by graphene quantum dots as a defect layer. We use the transfer matrix method^39,40^ to study the effect of controlling parameters such as Rabi-frequency, relative phase, angular momentum, and azimuthal phase of the applied fields on behavior of the Hartman effect. Transmission behavior of the weak probe field is also discussed to find the physical mechanisms of the obtained results.

In “Pulse propagation in 1DPC”, the density matrix equation of motions for the graphene quantum dots and propagation equation of a light pulse in 1DPC via the transfer matrix method (TMM) are introduced. The results are presented in “Results and discussion”, and finally the paper is concluded in “Conclusion”.

## Pulse propagation in 1DPC

**Figure 1:**
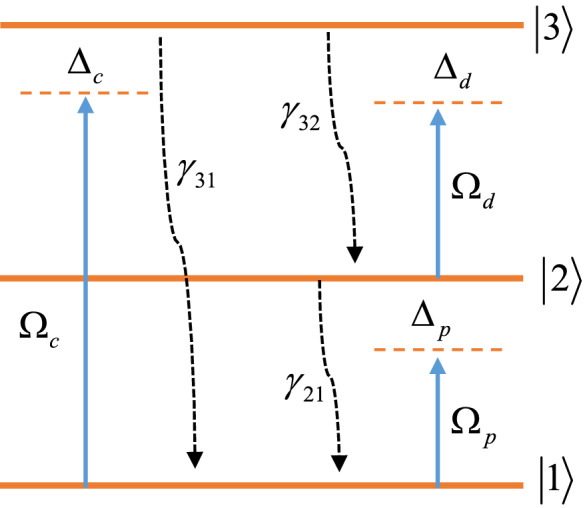
Diagram of a GQD.

Consider a quarter-wave stack with $$\left( {AB} \right)^{2} ADA\left( {BA} \right)^{N}$$ and $$N = 10$$. In this proposal A and B layers are considered as $${\text{SiO}}_{2}$$ and $${\text{TiO}}_{2}$$ with refractive indices 1.47 and 2.28, respectively^41,42^. The optical path length of layers are $$n_{{{\text{SiO}}_{2} }} d_{{_{{{\text{SiO}}_{2} }} }} = n_{{_{{{\text{TiO}}_{2} }} }} d_{{_{{{\text{TiO}}_{2} }} }} = \frac{{\lambda_{0} }}{4}$$, where $$\lambda_{0} = 500{\kern 1pt} \;{\text{nm}}$$ is the central wavelength of the applied probe field. The optical path length of the defect layer is $$n_{D} d_{D} = \frac{{\lambda_{0} }}{2}$$. In this study the defect layer of the 1DPC is doped by the ensemble of the nitrogen graphene quantum dots (GQDs). The proposed GQDs behave as three-level ladder-type quantum system^43^. As depicted in Fig. 1, the lower level, intermediate level and the upper level are labeled by $$\left| 1 \right\rangle$$, $$\left| 2 \right\rangle$$, and $$\left| 3 \right\rangle$$. A coupling laser field with Rabi-frequency $$\Omega_{c} = {{E_{c} \mu_{13} } \mathord{\left/ {\vphantom {{E_{c} \mu_{13} } {2\hbar }}} \right. \kern-0pt} {2\hbar }}$$ drives the transition $$\left| 1 \right\rangle \leftrightarrow \left| 3 \right\rangle$$, while another driving laser field with Rabi-frequency $$\Omega_{d} = {{E_{d} \mu_{23} } \mathord{\left/ {\vphantom {{E_{d} \mu_{23} } {2\hbar }}} \right. \kern-0pt} {2\hbar }}$$ applies to the transition $$\left| 2 \right\rangle \leftrightarrow \left| 3 \right\rangle$$. Furthermore, a weak tunable probe field with Rabi-frequency $$\Omega_{p} = {{E_{p} \mu_{12} } \mathord{\left/ {\vphantom {{E_{p} \mu_{12} } {2\hbar }}} \right. \kern-0pt} {2\hbar }}$$ couples levels $$\left| 1 \right\rangle$$ and $$\left| 2 \right\rangle$$. Here, $$E_{i} (i = c,$$ $$d,$$ $$p)$$ are the amplitudes of the corresponding fields, and $$\mu_{ij} (i,j = 1,$$ $$2,$$ $$3)$$ are the electric dipole moments of the corresponding transition. The Rabi-frequencies are complex quantities, so the respected phase of the applied fields are related to the Rabi-frequencies via relations $$\Omega_{p} = \left| {\Omega_{p} } \right|e^{{ - i\varphi_{p} }} ,$$ $$\Omega_{d} = \left| {\Omega_{d} } \right|e^{{ - i\varphi_{d} }}$$, and $$\Omega_{c} = \left| {\Omega_{c} } \right|e^{{ - i\varphi_{c} }}$$. Here, $$\varphi_{i} (i = p,$$ $$d,$$ $$c)$$ are the phase of the applied fields. Redefining the density matrix element as $$\rho_{12} = \tilde{\rho }_{12} e^{{ - i\varphi_{p} }}$$, $$\rho_{23} = \tilde{\rho }_{23} e^{{ - i\varphi_{d} }}$$, $$\rho_{13} = \tilde{\rho }_{13} e^{{ - i(\varphi_{d} + \varphi_{c} )}}$$, $$\rho_{ii} = \tilde{\rho }_{ii} (i = 1,$$ $$2,$$ $$3),$$ and using the Liouville equation, the density matrix equation of motions are obtained as^44,45^

1 $$\begin{aligned} \dot{\tilde{\rho }}_{22} = & i\Omega_{p} (\tilde{\rho }_{12} - \tilde{\rho }_{21} ) - i\Omega_{d} (\tilde{\rho }_{23} - \tilde{\rho }_{32} ) - \gamma_{21} \tilde{\rho }_{22} \\ & + \gamma_{32} \tilde{\rho }_{33} , \\ \dot{\tilde{\rho }}_{33} = & i\Omega_{d} (\tilde{\rho }_{23} - \tilde{\rho }_{32} ) + i\Omega_{c} e^{ - i\Delta \varphi } \tilde{\rho }_{13} - i\Omega_{c} e^{i\Delta \varphi } \tilde{\rho }_{31} \\ & - (\gamma_{21} + \gamma_{32} )\tilde{\rho }_{33} , \\ \dot{\tilde{\rho }}_{21} = & - i\Omega_{p} (\tilde{\rho }_{22} - \tilde{\rho }_{11} ) - i\Omega_{c} e^{ - i\Delta \varphi } \tilde{\rho }_{23} + i\Omega_{d} \tilde{\rho }_{31} \\ & - (i\Delta_{p} - \Gamma_{21} )\tilde{\rho }_{21} , \\ \dot{\tilde{\rho }}_{31} = & - i\Omega_{p} \tilde{\rho }_{32} - i\Omega_{c} e^{ - i\Delta \varphi } (\tilde{\rho }_{33} - \tilde{\rho }_{11} ) + i\Omega_{d} \tilde{\rho }_{21} \\ & - (i(\Delta_{p} + \Delta_{d} ) - \Gamma_{31} )\tilde{\rho }_{31} , \\ \dot{\tilde{\rho }}_{32} = & - i\Omega_{d} (\tilde{\rho }_{33} - \tilde{\rho }_{11} ) + i\Omega_{c} e^{ - i\Delta \varphi } \tilde{\rho }_{21} + i\Omega_{p} \tilde{\rho }_{31} \\ & - (i\Delta_{d} - \Gamma_{32} )\tilde{\rho }_{32} , \\ \end{aligned}$$

The relative phase of the applied fields define as $$\Delta \varphi = \varphi_{c} - \varphi_{d} - \varphi_{p}$$. In addition, the frequency detunings of the probe, driving and coupling fields are $$\Delta_{p} = \omega_{p} - \omega_{12}$$, $$\Delta_{d} = \omega_{d} - \omega_{23}$$, and $$\Delta_{c} = \omega_{c} - \omega_{13}$$, respectively. Here, $$\omega_{i} (i = p,$$ $$d,$$ $$c)$$ indicate the frequencies of the corresponding applied fields. The frequencies are selected in a way that the central frequencies satisfy the relations $$\omega_{c} = \omega_{d} + \omega_{p}$$ and $$\Delta_{c} = \Delta_{d} + \Delta_{p}$$. The parameters $$\gamma_{ij}$$ and $$\Gamma_{ij}$$ are the spontaneous decay rates and dephasing broadenings from level $$\left| i \right\rangle$$ to level $$\left| j \right\rangle$$, respectively. The linear susceptibility defines as

2 $$\chi = \frac{{2M\left| {\vec{\mu }_{21} } \right|^{2} }}{{\varepsilon_{0} \hbar \Omega_{p} }}\rho_{21} ,$$

where $M$ and $\varepsilon_{0}$ are the density of GQDs and the permittivity of free space with the plank constant $\hbar$. The density matrix Eq. (1) should be solved in steady state condition to obtain the real and imaginary parts of $\chi$ and consequently the refractive index $n$ by using the equation $n^{2} = \varepsilon_{B} + \chi (\omega )$. Here, $\varepsilon_{B}$ is the electric permittivity of the background medium. Thus, the absorptive and dispersive properties of the defect layer can be controlled by manipulating the parameters such as Rabi-frequencies, detunings and relative phase of applied fields.

Now, a probe pulse with a central frequency $\omega_{0}$ is normally incident upon the photonic crystal. In general, the electric (or magnetic) fields at two positions $z$ and $z + \Delta z$ in the same layer are related by a transfer matrix $M_{j} (\Delta z,\omega )$ as^39^

3 $$\psi_{j} (z + \Delta z,\omega ) = M_{j} (\Delta z,\omega )\psi_{j} (z,\omega )$$

where $\psi_{j} (z + \Delta z,\omega )$ and $\psi_{j} (z,\omega )$ are the exited and entered electric (or magnetic) fields, respectively. The transfer matrix $M_{j} (\Delta z,\omega )$ defines as

4 $$\begin{aligned} & M_{j} (\Delta z,\omega ) \\ \quad = \left( {\begin{array}{*{20}c} {\cos [\frac{\omega }{c}n_{j} (\omega )\Delta z]} & {\frac{1}{{n_{j} (\omega )}}\sin \left[ {\frac{\omega }{c}n_{j} (\omega )\Delta z} \right]} \\ { - n_{j} (\omega )\sin [\frac{\omega }{c}n_{j} (\omega )\Delta z]} & {\cos [\frac{\omega }{c}n_{j} (\omega )\Delta z]} \\ \end{array} } \right), \\ \end{aligned}$$

where $n_{j} (\omega )$ is the refractive index of $j$th layer. In the following discussion, we introduce a matrix as $\prod\nolimits_{j = 1}^{N} {M_{j} (d_{j} ,\omega )}$ that relates the entered and exited beams of light in a photonic crystal. This term represents as

5 $$X_{N} (\omega ) = \prod\limits_{j = 1}^{N} {M_{j} } (d_{j} ,\omega ) = \left( {\begin{array}{*{20}c} {x_{11} } & {x_{12} } \\ {x_{21} } & {x_{22} } \\ \end{array} } \right),$$

where $x_{ij} (i,j = 1,2)$ are the elements of this matrix. This can be used to obtain the reflection $r(\omega )$ and transmission $t(\omega )$ coefficients of a monochromatic wave pulse of frequency $\omega$ as^39,46^

6 $$r(\omega ) = \frac{{[x_{22} (\omega ) - n_{s} x_{11} (\omega )] - i[n_{s} x_{12} (\omega ) + x_{21} (\omega )]}}{{[x_{22} (\omega ) + n_{s} x_{11} (\omega )] - i[n_{s} x_{12} (\omega ) - x_{21} (\omega )]}},$$

and

7 $$t(\omega ) = \frac{2}{{[x_{22} (\omega ) + n_{s} x_{11} (\omega )] - i[n_{s} x_{12} (\omega ) - x_{21} (\omega )]}}.$$

Here, $n_{s}$ indicates the refractive index of subtract. In order to separate the real and imaginary parts of the $r(\omega )$ and $t(\omega )$, we write them in the following form $t(\omega ) = \left| {t(\omega )} \right|\exp [i\phi_{t} (\omega )]$ and $r(\omega ) = \left| {r(\omega )} \right|\exp [i\phi_{r} (\omega )]$, where the real functions $\phi_{r,t} (\omega )$ are the phases of the transmission and reflection coefficients, respectively. The phase time for transmitted and reflected pulses are obtained by the relation $\tau_{r,t} (\omega ) = \frac{{\partial \phi_{r,t} }}{\partial \omega }$^46,47^. It is possible to calculate the transmitted phase shift by using the method that is described in reference^48^. In this method, the total phase, which is accumulated by propagating light inside the medium, is considered as $\phi_{t} = \tan^{ - 1} ({y \mathord{\left/ {\vphantom {y x}} \right. \kern-0pt} x}) \pm m\pi$. In this equation, the integer *m* is defined according to the fact that $\phi_{t} (\omega )$ is a monotonic increasing function. In a condition $\omega \to 0$ it is acceptable to take $m = 0$. Then the transmitted and reflected phase times are obtained from^46^

8 $$\begin{aligned} \tau_{t} (\omega ) = & \frac{{\partial \phi_{t} }}{\partial \omega } = \frac{1}{{\left| {t(\omega )} \right|^{2} }} \\ & \times \left( {{\text{Re}} [t(\omega )]\frac{{\partial {\text{Im}} [t(\omega )]}}{\partial \omega } - {\text{Im}} [t(\omega )]\frac{{\partial {\text{Re}} [t(\omega )]}}{\partial \omega }} \right), \\ \end{aligned}$$

and

9 $$\begin{aligned} \tau_{r} (\omega ) = & \frac{{\partial \phi_{r} }}{\partial \omega } = \frac{1}{{\left| {r(\omega )} \right|^{2} }} \\ & \times \left( {{\text{Re}} [r(\omega )]\frac{{\partial {\text{Im}} [r(\omega )]}}{\partial \omega } - {\text{Im}} [r(\omega )]\frac{{\partial {\text{Re}} [r(\omega )]}}{\partial \omega }} \right), \\ \end{aligned}$$

We assume that the coupling field $\Omega_{c}$ carries the orbital angular momentum $(\hbar l)$ along the propagation axis z^32^. Therefore, the Rabi-frequency of this field can be rewritten as

10 $$\Omega_{c} = \left| {\Omega_{c} } \right|\exp (il\Phi ),$$

where $\Phi$ and $l$ are the azimuthal phase and the related OAM, respectively.

For a Laguerre–Gaussian doughnut beam, we write

11 $$\left| {\Omega_{c} } \right| = E_{c} \left( {\frac{r}{{\omega_{c} }}} \right)^{\left| l \right|} \exp \left( { - \frac{{r^{2} }}{{\omega_{c}^{2} }}} \right),$$

where $r$, $\omega_{c}$ and $E_{c}$ represent the distance from the vortex core (cylindrical radius), beam waist and strength of the vortex beam, respectively. We assume that the two other fields have no orbital momentum, and thus Rabi-frequencies can be defined as $\Omega_{d} = \left| {\Omega_{d} } \right|$ and $\Omega_{p} = \left| {\Omega_{p} } \right|$.

## Results and discussion

**Figure 2:**
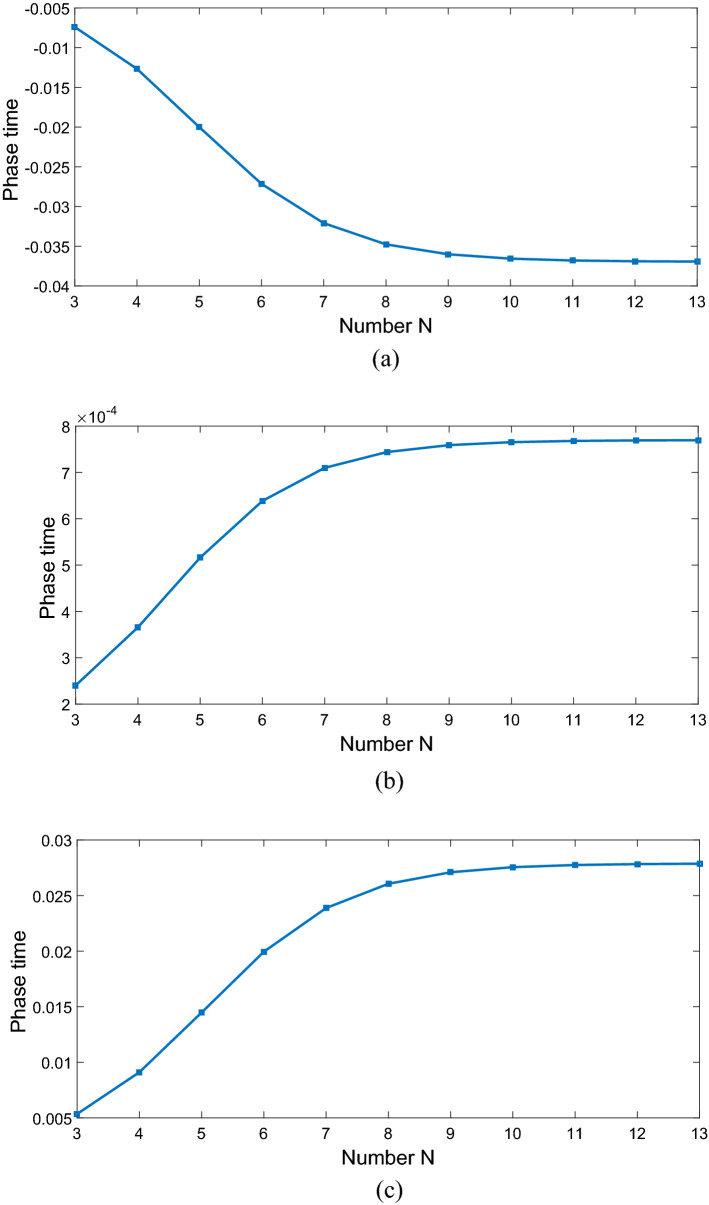
The Hartman effect in a 1DPC's with structure $\left( {AB} \right)^{2} ADA\left( {BA} \right)^{N}$ for (**a**) $\Omega_{c} = 0.6\gamma ,$ $\Omega_{d} = 0.9\gamma ,$ $\Delta \varphi = 0$, (**b**) $\Omega_{c} = 0.6\gamma ,$ $\Omega_{d} = 5\gamma ,$ $\Delta \varphi = 0$, (**c**) $\Omega_{c} = 0.6\gamma ,$ $\Omega_{d} = 0.9\gamma ,$ and $\Delta \varphi = \frac{\pi }{2}$. Here, $D$ denotes the existence of QD defect layer. Other parameters are $\gamma_{21} = \gamma_{31} = \gamma_{32} = \gamma = 1\;{\text{mev,}}$ $\Gamma_{21} = \Gamma_{31} = \Gamma_{32} = 5\gamma ,$ $\Omega_{p} = 0.2\gamma$, and $\Delta_{d} = 0.$

**Figure 3:**
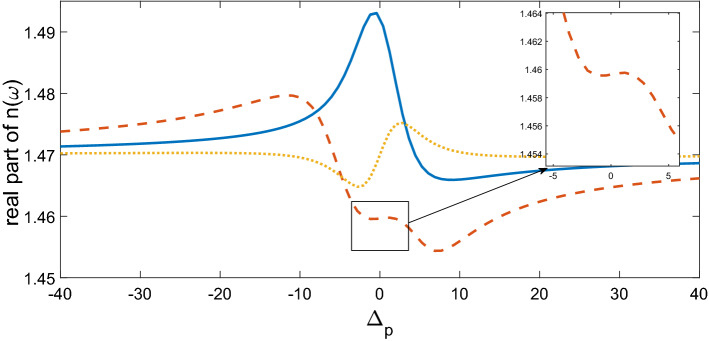
The real part of refractive index for $\Omega_{c} = 0.6\gamma ,$ $\Omega_{d} = 0.9\gamma ,$ $\Delta \varphi = 0$ (solid line), $\Omega_{c} = 0.6\gamma ,$ $\Omega_{d} = 5\gamma ,$ $\Delta \varphi = 0$ (dashed line), $\Omega_{c} = 0.6\gamma ,$ $\Omega_{d} = 0.9\gamma ,$ and $\Delta \varphi = \frac{\pi }{2}$ (dotted line). Other parameters are $\gamma_{21} = \gamma_{31} = \gamma_{32} = \gamma = 1\;{\text{mev}},$ $\Gamma_{21} = \Gamma_{31} = \Gamma_{32} = 5\gamma ,$ $\Omega_{p} = 0.2\gamma$, and $\Delta_{d} = 0.$

**Figure 4:**
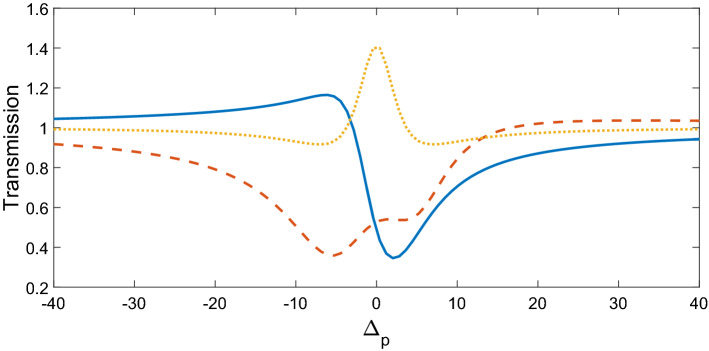
Transmission coefficient of probe field versus normalized probe filed detuning for $\Omega_{c} = 0.6\gamma ,$ $\Omega_{d} = 0.9\gamma ,$ $\Delta \varphi = 0$ (solid line), $\Omega_{c} = 0.6\gamma ,$ $\Omega_{d} = 5\gamma ,$ $\Delta \varphi = 0$ (dashed line), $\Omega_{c} = 0.6\gamma ,$ $\Omega_{d} = 0.9\gamma ,$ and $\Delta \varphi = \frac{\pi }{2}$ (dotted line).

**Figure 5:**
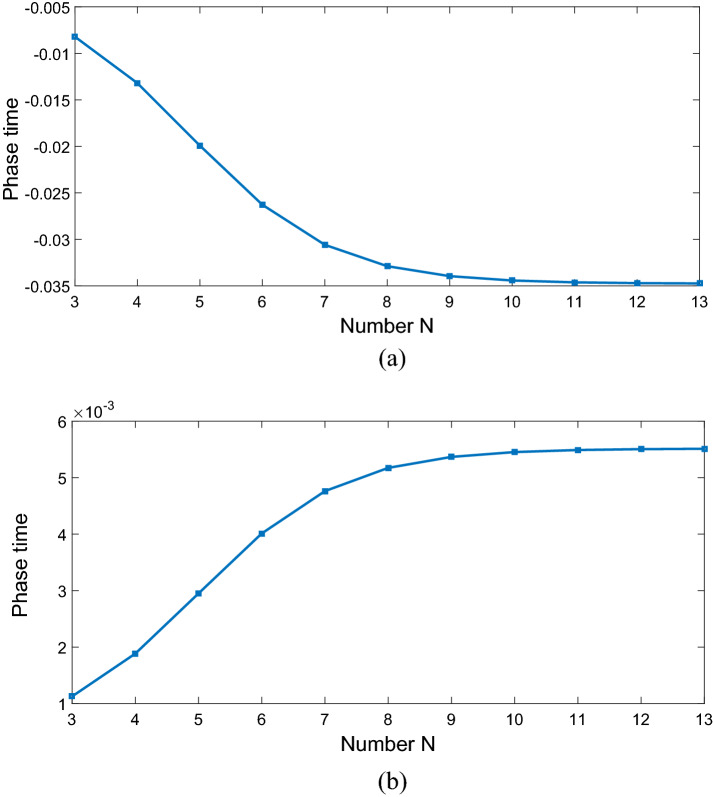
The Hartman effect in a 1DPC's with structure $\left( {AB} \right)^{2} ADA\left( {BA} \right)^{N}$ when only one control field $\Omega_{c}$ has an optical vortex with OAM numbers $l = 2$ (**a**), $l = - 2$ (**b**). Here $\Delta \varphi = 0,$ $\Omega_{p} = 0.2\gamma ,$ $\Omega_{d} = 0.9\gamma ,$ $\omega_{c} = 1\;\upmu {\text{m,}}$ $\Phi = {\pi \mathord{\left/ {\vphantom {\pi 4}} \right. \kern-0pt} 4},$ $E_{c} = \gamma$, and $r = 1.$

**Figure 6:**
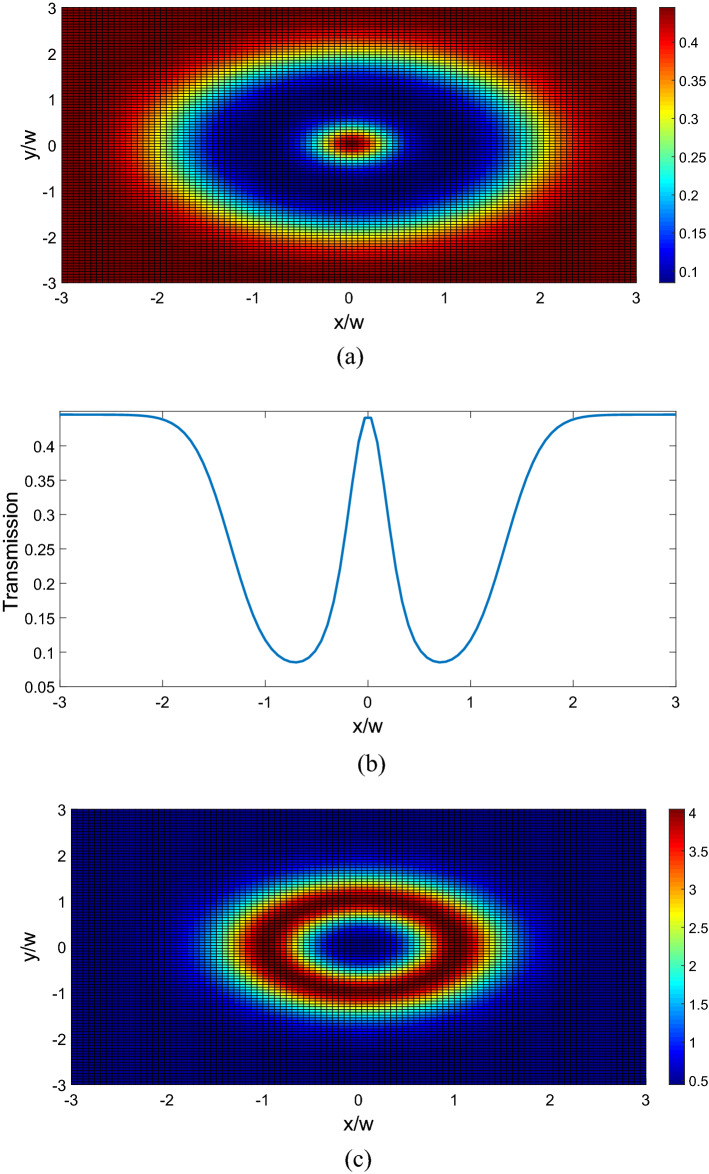

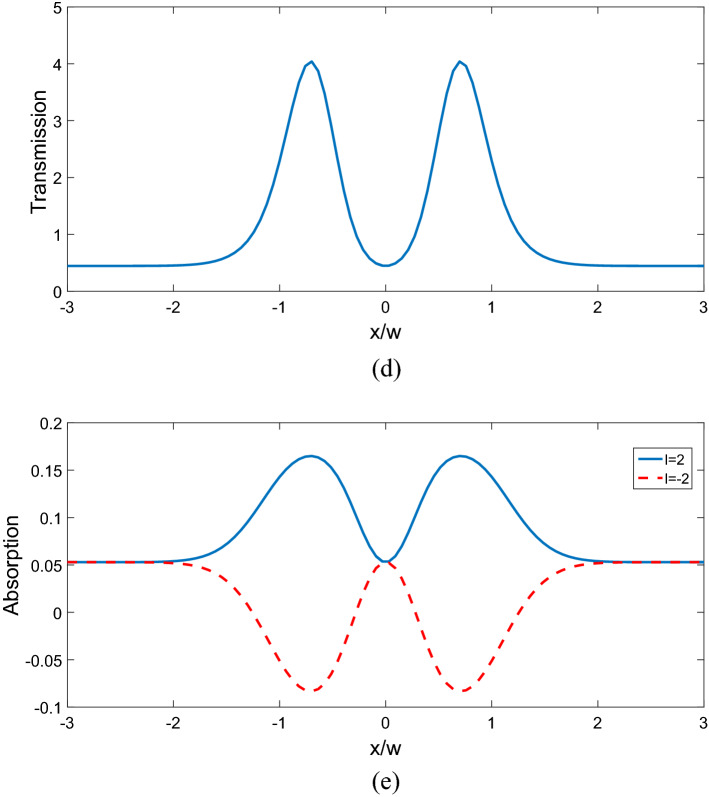
Three dimensional probe field transmission in transverse (*x*–*y*) plane (**a**), probe field transmission as a function of normalized position $${x \mathord{\left/ {\vphantom {x \omega }} \right. \kern-0pt} \omega }$$ for $$l = 2$$ (**b**). Three dimensional probe field transmission in transverse (*x*–*y*) plane (**c**), probe field transmission as a function of normalized position $${x \mathord{\left/ {\vphantom {x \omega }} \right. \kern-0pt} \omega }$$ for $$l = - 2$$ (**d**). Probe field absorption as a function of normalized position $${x \mathord{\left/ {\vphantom {x \omega }} \right. \kern-0pt} \omega }$$ for $$l = 2$$ and $$l = - 2$$ (**e**). Other selected parameters are $$\Delta \varphi = 0,$$ $$\Omega_{p} = 0.2\gamma ,$$$$\Omega_{d} = 0.9\gamma ,$$ $$\omega_{c} = 1\;\upmu {\text{m,}}$$ $$\Phi = {\pi \mathord{\left/ {\vphantom {\pi 4}} \right. \kern-0pt} 4},$$$$E_{c} = \gamma$$, and $$\Delta_{p} = 0.$$

**Figure 7:**
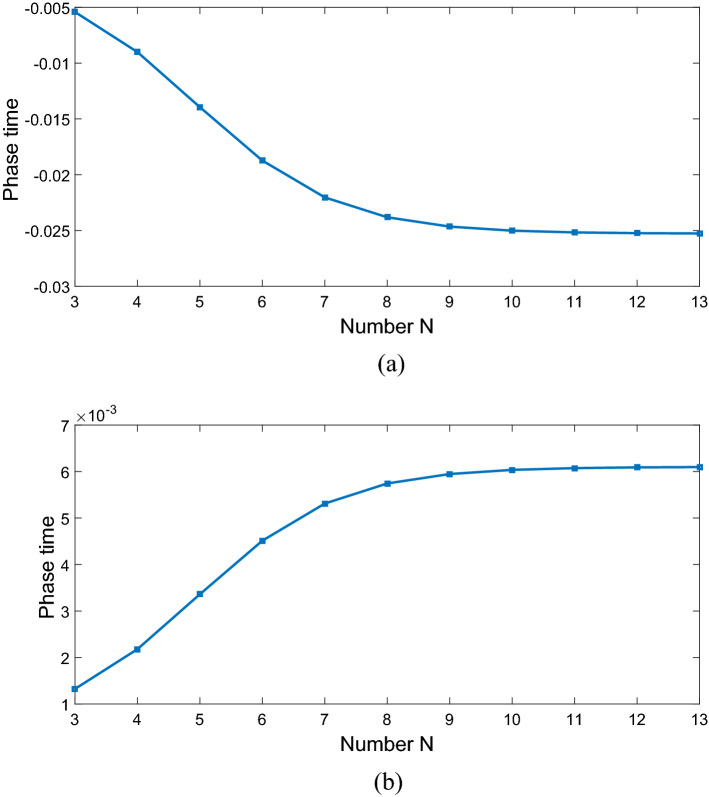
The Hartman effect in a 1DPC's with structure $\left( {AB} \right)^{2} ADA\left( {BA} \right)^{N}$ when only one control field $\Omega_{c}$ has an optical vortex with azimuthal phase $\Phi = 0$ (**a**), $\Phi = \frac{2\pi }{3}$ (**b**). Other parameters are $\Delta \varphi = 0,$ $\Omega_{p} = 0.2\gamma ,$ $\Omega_{d} = 0.9\gamma ,$ $\omega_{c} = 1\;\upmu {\text{m,}}$ $l = 2,$ $r = 1$, and $E_{c} = \gamma$.

**Figure 8:**
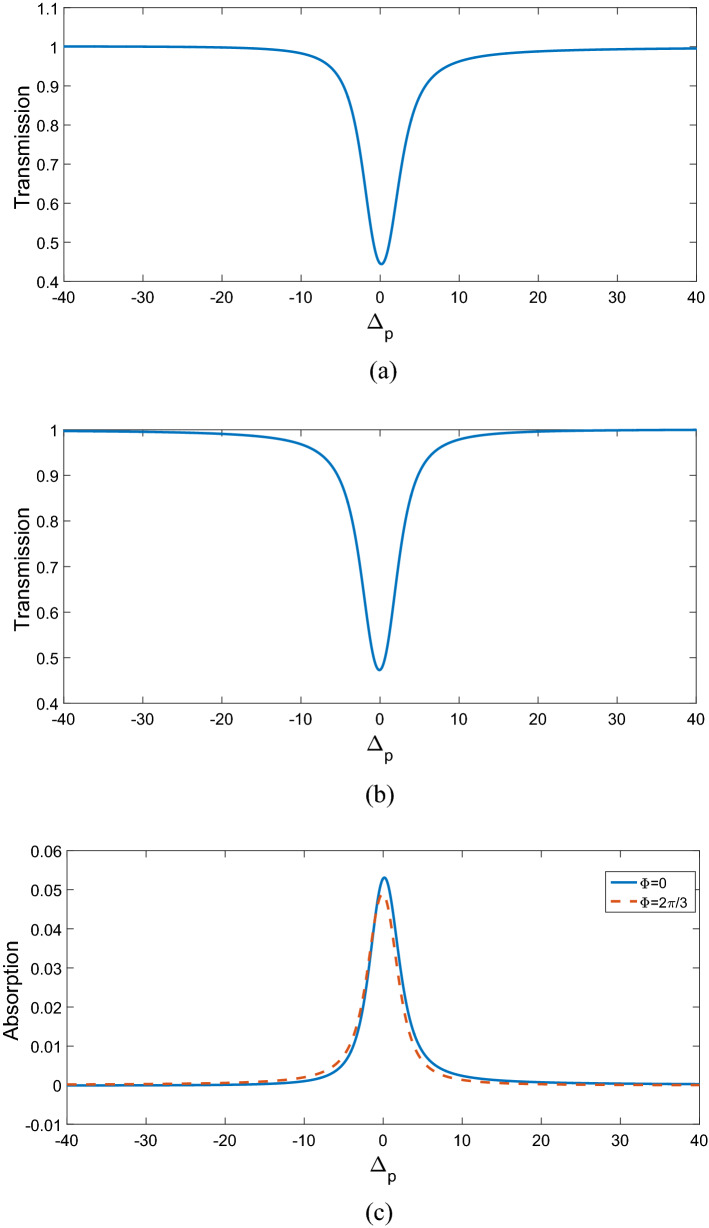
Transmission coefficient of probe field versus normalized probe filed detuning for $\Phi = 0$ (**a**), $\Phi = \frac{2\pi }{3}$ (**b**). Absorption coefficient of probe field versus normalized probe filed detuning for $\Phi = 0$ and $\Phi = \frac{2\pi }{3}$ (**c**). Other parameters are same as in Fig. 7.

Now, we investigate the effects of various controlling parameters including Rabi-frequencies, relative phase, angular momentum and azimuthal phase of the applied fields on transmitted phase time. In order to simplify the results, we chose the spontaneous emission $$\gamma_{21} = \gamma = 1$$ meV and all the other parameters are normalized by $$\gamma$$. Also, we assume all the GQDs are initially in ground state, i.e. $$\rho_{11} (0) = 1$$ and $$\rho_{ij} (0) = 0(i,j = 1,2,3).$$ Furthermore, the central wavelength of the probe laser field is considered as $$\lambda_{0} = 500\;{\text{nm}}$$. In addition, $$\left| {\vec{\mu }_{21} } \right| = 9.6 \times 10^{ - 29} {\text{C}}\;{\text{m}},$$$$M = 5 \times 10^{15} \;{\text{cm}}^{ - 3} ,$$ and $$\Omega_{p} = 0.2\gamma .$$ Figure 2 shows the dependence of the transmitted phase time on periodic number N of a 1DPC with a structure $$\left( {AB} \right)^{2} ADA\left( {BA} \right)^{N}$$. For $$\Omega_{c} = 0.6\gamma ,$$ $$\Omega_{d} = 0.9\gamma ,$$ and $$\Delta \varphi = 0,$$ the transmitted phase time remains negative for all values of N, which corresponds to the negative Hartman effect (Fig. 2a). By increasing $$\Omega_{d}$$ from $$0.9\gamma$$ to $$5\gamma$$, the transmitted phase time becomes positive and reaches to a constant value, which implies a positive Hartman effect (Fig. 2b). Note that for the same parameters presented in Fig. 2a, just by changing the relative phase of the applied fields from $$\Delta \varphi = 0$$ to $$\Delta \varphi = \frac{\pi }{2}$$, again the negative phase time changes to positive as depicted in Fig. 2c. These results show that the phase time is completely sensitive to the amplitude of the coupling field and the relative phase of the applied fields. Thus, the Hartman effect can be tuned from negative to positive just by manipulating the amplitude of the driving field and the relative phase of applied fields. We emphases that negative (positive) Hartman effect is corresponding to superluminal (subluminal) light propagation. Thus, the superluminal light propagation changes to subluminal light propagation just by adjusting the parameters $$\Omega_{d}$$ and $$\Delta \varphi$$. In order to justify the physical mechanisms, we plot the real part of the effective refractive index as a function of probe field detuning in Fig. 3, i.e. $$n^{2} = \varepsilon_{B} + \chi (\omega )$$. By increasing the Rabi-frequency of the driving field, the negative slope of the dispersion (solid line) changes to positive one (dashed line). In addition, by switching $$\Delta \varphi$$ from zero to $$\frac{\pi }{2}$$, the slope of dispersion curve changes from negative to positive (dotted line). Note that negative (positive) slope of the dispersion curve is corresponding to superluminal (subluminal) light propagation as depicted in Fig. 2. These results are confirmed by the curve of transmitted probe field as a function of $$\Delta_{p}$$(Fig. 4). We find that for $$\Omega_{d} = 0.9\gamma$$ a dip appears in transmission curve, while it changes to a peak for $$\Omega_{d} = 5\gamma$$. This is also repeated for $$\Omega_{d} = 0.9\gamma$$ and $$\Delta \varphi = \frac{\pi }{2}$$ as depicted in dotted line. Now, we consider the coupling field ($$\Omega_{c}$$) as an optical vortex light that carries OAM. In this case, two other controlling parameters including OAM and azimuthal phase appear that can be used to control the Hartman effect. In Fig. 5a, for $$l = 2$$ the transmitted phase time leads to a negative constant corresponding to superluminal light propagation through the 1DPC. However, as shown in Fig. 5b, for $$l = - 2$$, this changes to a positive one corresponding to subluminal light propagation. In Fig. 6a, the three dimensional transmission pattern of the probe field as a function of the transverse $$(x - y)$$ directions is plotted for $$l = 2$$. It is obvious that the 45% of the probe field is transmitted around $$x = 0$$ and $$y = 0$$. This is confirmed by the transmission curve of probe field as a function of $${x \mathord{\left/ {\vphantom {x \omega }} \right. \kern-0pt} \omega }$$ as depicted in Fig. 6b. It is worth noting that by changing $$l$$ of the controlling field from 2 to − 2, the probe field transmission remains unchanged at the vicinity of $$x = 0$$ and $$y = 0$$. This is an important result, where by manipulating the $$l$$ from 2 to − 2, the negative Hartman effect changes to positive while the probe field transmission remains unchanged. By comparing the results of Figs. 4 and 6, one can notice that by selecting $$\Omega_{c}$$ as an optical vortex light, we can obtain better results in converting negative Hartman effect to positive than an ordinary laser field. In Fig. 6e, the absorption spectrum of the probe field is plotted. It can be seen that the absorption at the vicinity of $$x = 0$$ and $$y = 0$$ is low and converting the amount of $$l$$ from 2 to − 2 does not effect it. This is the main result for neglecting the imaginary parts of $${\text{SiO}}_{2}$$ and $${\text{TiO}}_{2}$$ refractive indices and taking only the real parts of them. Another important parameter is the impact of azimuthal phase on the Hartman effect. Figure 7a demonstrates that for $$\Phi = 0$$, the phase time delay reaches to a negative constant corresponding to negative Hartman effect. Nevertheless, as demonstrated in Fig. 7b, by setting the azimuthal phase to $$\Phi = \frac{2\pi }{3}$$, the transmitted phase time tends to a positive constant implying positive Hartman effect. Thus, the Hartman effect can be manipulated just by adjusting the azimuthal phase of the controlling field. Again, in order to investigate the effect of the azimuthal phase of the controlling field on the transmission profile of the probe field (Fig. 8), we plot the transmission as a function of the probe field detuning $$\Delta_{p}$$. It is proven that the transmission around $$\Delta_{p} = 0$$ is the same for $$\Phi = 0$$ and $$\Phi = \frac{2\pi }{3}$$. We emphasis that for a vortex beam any change of the orbital angular momentum number and azimuthal phase does not affect the probe field transmission while the Hartman effect is altered. Figure 8c shows the absorption spectrum of the probe field. It is apparent that the absorption at the $$\Delta_{p} = 0$$ is so weak and it remains the same by changing $$\Phi$$ from zero to $$\frac{2\pi }{3}$$. Again this proves that neglecting the imaginary parts of the $${\text{SiO}}_{2}$$ and $${\text{TiO}}_{2}$$, refractive indices does not influence the obtained result for Hartman effect.

## Conclusion

The Hartman effect in a 1DPC doped with graphene quantum dots is investigated. It is shown that the phase time delay tends to a constant value by increasing the number of the photonic crystal layers. Amplitude of the controlling field as well as the relative phase of the applied fields affect the presented Hartman effect. Orbital angular momentum and azimuthal phase can also be utilized to manipulate the Hartman effect if one controlling field is assumed to be a vortex beam. We demonstrate that while the Hartman effect is converted from negative to positive, the probe field transmission remains unchanged. This makes the system applicable experimentally. Thus, in this paper we have shown that by using a photonic crystal with a defect layer doped with graphene quantum dots, the Hartman effect can be controlled not only by conventional methods as manipulating the Rabi-frequencies and relative phases of the applied fields but also by using a vortex beam as a controlling field. In the lateral case, two new parameters i.e. the orbital angular momentum $(l)$ and the azimuthal phase $(\Phi )$ can be utilized as controlling parameters of the Hartman effect. We again emphases that switching the Hartman effect from negative to positive and vice versa is a very effective method in creating and controllable subluminal and superluminal light propagation.

## Data Availability

The datasets used and/or analysed during the current study are available from the corresponding author on reasonable request.
